# Thanatin Impairs Lipopolysaccharide Transport Complex Assembly by Targeting LptC–LptA Interaction and Decreasing LptA Stability

**DOI:** 10.3389/fmicb.2020.00909

**Published:** 2020-05-13

**Authors:** Elisabete C. C. M. Moura, Tiago Baeta, Alessandra Romanelli, Cedric Laguri, Alessandra M. Martorana, Emanuela Erba, Jean-Pierre Simorre, Paola Sperandeo, Alessandra Polissi

**Affiliations:** ^1^Dipartimento di Scienze Farmacologiche e Biomolecolari, Università degli Studi di Milano, Milan, Italy; ^2^Université Grenoble Alpes, CNRS, CEA, IBS, Grenoble, France; ^3^Dipartimento di Scienze Farmaceutiche, Università degli Studi di Milano, Milan, Italy

**Keywords:** bacterial cell wall, lipopolysaccharide, Lpt system, thanatin, antimicrobial peptides, BACTH technique, NMR

## Abstract

The outer membrane (OM) of Gram-negative bacteria is a highly selective permeability barrier due to its asymmetric structure with lipopolysaccharide (LPS) in the outer leaflet. In *Escherichia coli*, LPS is transported to the cell surface by the LPS transport (Lpt) system composed of seven essential proteins forming a transenvelope bridge. Transport is powered by the ABC transporter LptB_2_FGC, which extracts LPS from the inner membrane (IM) and transfers it, through LptC protein, to the periplasmic protein LptA. Then, LptA delivers LPS to the OM LptDE translocon for final assembly at the cell surface. The Lpt protein machinery operates as a single device, since depletion of any component leads to the accumulation of a modified LPS decorated with repeating units of colanic acid at the IM outer leaflet. Moreover, correct machine assembly is essential for LPS transit and disruption of the Lpt complex results in LptA degradation. Due to its vital role in cell physiology, the Lpt system represents a good target for antimicrobial drugs. Thanatin is a naturally occurring antimicrobial peptide reported to cause defects in membrane assembly and demonstrated *in vitro* to bind to the N-terminal β-strand of LptA. Since this region is involved in both LptA dimerization and interaction with LptC, we wanted to elucidate the mechanism of inhibition of thanatin and discriminate whether its antibacterial effect is exerted by the disruption of the interaction of LptA with itself or with LptC. For this purpose, we here implemented the Bacterial Adenylate Cyclase Two-Hybrid (BACTH) system to probe *in vivo* the Lpt interactome in the periplasm. With this system, we found that thanatin targets both LptC–LptA and LptA–LptA interactions, with a greater inhibitory effect on the former. We confirmed *in vitro* the disruption of LptC–LptA interaction using two different biophysical techniques. Finally, we observed that in cells treated with thanatin, LptA undergoes degradation and LPS decorated with colanic acid accumulates. These data further support inhibition or disruption of Lpt complex assembly as the main killing mechanism of thanatin against Gram-negative bacteria.

## Introduction

The emergence and spread of multidrug resistant pathogens pose an alarming threat to human and animal health worldwide. The old classes of antibiotics are becoming ineffective at killing an increasing number of pathogens and the decline in the discovery and development of new drugs, experienced in recent years, is seriously eroding the ability of clinicians to control infectious diseases, making the identification of new antimicrobial compounds with novel mechanisms of action an urgent need (Tacconelli et al., 2018). This situation is even more worrisome for Gram-negative pathogens since they are endowed with an asymmetric outer membrane (OM), surrounding the inner membrane (IM) and delimiting a peptidoglycan-containing periplasmic space, that protects them from harmful hydrophobic compounds such as antibiotics (Nikaido, 2003). The peculiar permeability barrier properties of the OM are conferred by the presence of a layer of tightly packed molecules of lipopolysaccharide (LPS) in its outer leaflet (Raetz and Whitfield, 2002; Silhavy et al., 2010). LPS consists of three covalently linked moieties: lipid A, the conserved hydrophobic anchor of the molecule in the membrane; a core oligosaccharide; and a somewhat variable polysaccharide chain, termed *O* antigen (Raetz and Whitfield, 2002). The biosynthesis of the lipid A-core domain takes place at the cytoplasmic side of the IM, whereas the assembly of mature LPS occurs at the periplasmic side of the IM, after flipping of the lipid A-core across the IM by the essential transporter MsbA (Polissi and Georgopoulos, 1996; Raetz and Whitfield, 2002; Doerrler et al., 2004).

**FIGURE 1:**
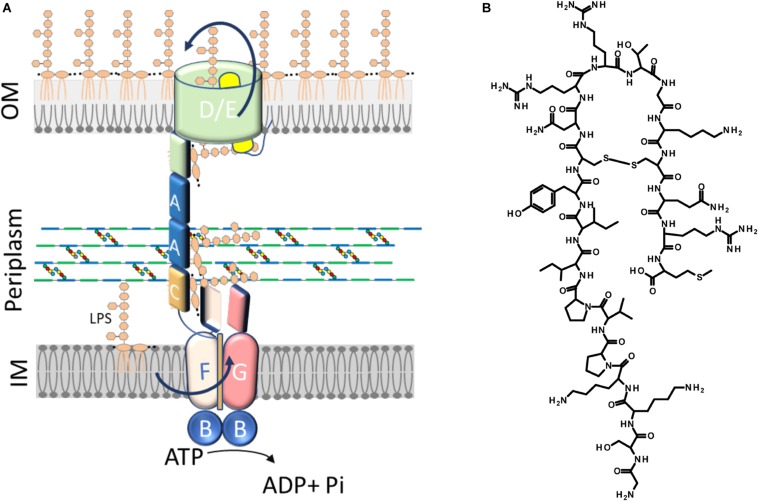
The Lpt machinery and thanatin. **(A)** The lipopolysaccharide transport system in *Escherichia coli* consists of a seven-protein complex organized in an inner membrane (IM) ABC transporter (LptB_2_FGC) and an outer membrane (OM) translocon (LptDE) connected by a periplasmic protein, LptA, that bridges the membranes. LptA is anchored to the IM through its interaction with LptC. The number of LptA molecules forming the bridge is not known. For clarity, only two molecules of LptA are depicted. **(B)** Structure of thanatin.

Translocation of LPS from the IM to the OM, across the periplasm, requires the activity of the LPS transport (Lpt) machinery. This assembly is a conserved multiprotein complex composed, in *Escherichia coli*, of seven essential proteins (LptA-G) that bridges the IM and OM (Wu et al., 2006; Sperandeo et al., 2007, 2008; Ruiz et al., 2008; Freinkman et al., 2012) (Figure 1A). The Lpt partners are organized in three sub-complexes, located in each cell envelope compartment (IM, periplasm, and OM), that interact with each other to allow the transport of LPS to the OM, shielding the hydrophobic moieties of lipid A in the hydrophilic environment of the periplasm (Sperandeo et al., 2008). At the IM, the ABC transporter LptB_2_FGC provides the energy for LPS extraction from the IM (Okuda et al., 2012; Li et al., 2019; Owens et al., 2019). The unconventional subunit LptC plays a dual role in the transporter, regulating the ATPase activity and providing the docking site for the periplasmic protein LptA at the membrane (Sperandeo et al., 2011; Owens et al., 2019). After extraction, LPS is transferred from LptC to LptA (Tran et al., 2010; Okuda et al., 2012), that then interacts at the OM with the periplasmic domain of LptD forming the bridge that connects the IM and OM (Okuda et al., 2016). LptA has the tendency to oligomerize *in vitro* (Suits et al., 2008; Merten et al., 2012; Santambrogio et al., 2013); however, the number of LptA monomers that constitute the Lpt bridge is still not known. At the OM, the translocon composed of the β-barrel protein LptD and the lipoprotein LptE receives LPS from LptA for its final assembly at the cell surface (Freinkman et al., 2011; Dong et al., 2014; Qiao et al., 2014). The interaction between the Lpt proteins is crucial in building a functional machinery (Sperandeo et al., 2011; Falchi et al., 2018) and is mediated by a conserved domain with a peculiar structural architecture (the β-jellyroll fold) shared by all the periplasmic domains of the Lpt proteins (LptF, LptG, LptC, LptA, and LptD) (Suits et al., 2008; Tran et al., 2010; Qiao et al., 2014). Alignment of the β-jellyroll folds of LptF, LptC, LptA, and LptD in a C-terminal-to-N-terminal arrangement is thought to allow the formation of a hydrophobic groove that spans the periplasm and accommodates the acyl chains of the LPS molecules during transport (Villa et al., 2013; Okuda et al., 2016; Sperandeo et al., 2019). Inhibition of bridge formation, as a consequence of Lpt protein depletion in conditional expression mutants or due to mutations that interfere with protein–protein interactions at any level in the system, results in cell growth arrest and blocking of Lpt, with accumulation of newly synthesized LPS in the IM and formation of membranous bodies in the periplasm (Wu et al., 2006; Sperandeo et al., 2007, 2008; Ruiz et al., 2008). Accumulated LPS molecules can be decorated at the periplasmic side of the IM by the addition of colanic acid units (Majdalani and Gottesman, 2005; Sperandeo et al., 2008, 2011). Overall, the Lpt mechanism mediated by the Lpt machinery has been compared to that of a PEZ candy dispenser, where a spring at the base of the dispenser loads the candy into the tube and pushes them up to the cap, which then opens to release them to the customer (Okuda et al., 2016). Interestingly, when the Lpt bridge is not properly assembled, LptA undergoes degradation, suggesting that the steady-state level of LptA in the cell, together with the appearance of colanic acid-modified LPS, are diagnostic of Lpt defects (Sperandeo et al., 2011).

Due to its relevance in Gram-negative bacteria cell physiology, LPS biogenesis can be considered a promising target for the development of novel antibacterial molecules. Potent inhibitors of the lipid A biosynthesis were identified in past studies and are continuously in development (Simpson and Trent, 2019). Moreover, two compounds targeting the MsbA-mediated IM translocation process have been recently reported (Ho et al., 2018; Zhang et al., 2018). However, the only inhibitor of LPS biogenesis to have entered, so far, Phase III trials is Murepavadin, a macrocyclic peptidomimetic selectively directed against *Pseudomonas aeruginosa* LptD (Srinivas et al., 2010; Lehman and Grabowicz, 2019). Unfortunately, the clinical trials have been suspended recently due to nephrotoxicity (Lehman and Grabowicz, 2019). Nevertheless, the identification of Murepavadin highlights the Lpt machinery as a good target for the discovery of molecules endowed with antibacterial activity.

Very recently, a screening strategy based on the yeast two-hybrid (YTH) system has allowed the isolation of a compound, IMB-881, that disrupts LptC–LptA interaction, exerting bactericidal activity against *E. coli* and other Enterobacterial species (Zhang et al., 2019).

Here we show the implementation of the Bacterial Adenylate Cyclase Two-Hybrid (BACTH) system (Karimova et al., 1998), based on the interaction-mediated reconstitution of the adenylate cyclase activity in *E. coli*, to allow the detection of LptC–LptA and LptA–LptA interactions in their native environment, the periplasm. We successfully reconstituted both interactions and exploited this system to more thoroughly investigate the effect of the antimicrobial peptide thanatin.

Thanatin is a 21-residue inducible cationic defense peptide isolated from the hemipteran insect *Podisus maculiventris*, that contains one disulfide bond and exhibits a broad range of antibacterial and antifungal activity (Fehlbaum et al., 1996) (Figure 1B).

Important new insights into thanatin’s mode of action against Gram-negative bacteria have been provided by a recent work showing that thanatin binds to *E. coli* LptA and LptD *in vivo* and *in vitro* (Vetterli et al., 2018). Accordingly, spontaneous thanatin-resistant mutants isolated in the same work share a single point mutation in the *lptA* gene, strongly indicating LptA as the major target of thanatin. Analysis of the nuclear magnetic resonance (NMR) structure of the LptA–thanatin complex reveals that the interaction occurs at the N-terminal β-strand of the β-jellyroll of LptA, region involved in LptA interaction with LptC and/or with another monomer of LptA (Suits et al., 2008; Freinkman et al., 2012). It has been thus speculated that thanatin might exert its antibacterial activity by interfering with the interactions established by LptA within the Lpt bridge (Vetterli et al., 2018). However, no evidence supporting this hypothesis has been published yet.

Our investigation provides more insights into thanatin’s mode of action against Gram-negative bacteria showing that it interferes with LptC–LptA interaction *in vivo*. Disruption of the Lpt protein bridge is further supported by LptA degradation and appearance of LPS modified by colanic acid in thanatin treated cells. The results of this work strongly validate the assembly of the Lpt machinery as a promising target for the development of a novel class of antibacterial or adjuvant drugs.

## Materials and Methods

### Bacterial Strains and Media

*Escherichia coli* strains and plasmids used in this study are listed in Table 1. AM604 genomic DNA was used as template for PCR and the XL1-Blue strain was used in all cloning steps. The strain MG1655 was used in the study of LptA stability and in the analysis of LPS profiles. BACTH assays were performed with the *E. coli* Δ*cya* strain BTH101 (Karimova et al., 1998; Ouellette et al., 2017). The strains M15/pREP4 and BL21(DE3) were used in the purification of LptC_24–191_ (Sperandeo et al., 2011) and LptA_*m*_ (Laguri et al., 2017), respectively. Bacteria were grown in Luria–Bertani (LB) medium (10 g/L tryptone, 5 g/L yeast extract, 10 g/L NaCl) or LB-agar medium (LB medium with 10 g/L agar). When required, antibiotics or inducer were added at the following concentrations: ampicillin at 100 μg/mL, spectinomycin at 50 μg/mL, isopropyl-β-D-thiogalactopyranoside (IPTG) at 0.5 mM.

**TABLE 1:** *Escherichia coli* strains and plasmids.

Strain or plasmid	Relevant genotype or description		Source or references
Strains			
MG1655	K-12, F^–^ λ^–^ *ilvG^–^ rfb-50 rph-1*		Blattner et al., 1997
AM604	MC4100 ara ^+^		Wu et al., 2006
AS19	*E. coli* strain B, hyperpermeable strain		Sekiguchi and Iida, 1967
NR698	*imp4213*		Ruiz et al., 2005
XL1-Blue	*recA1 endA1 gyrA96* (Nal^*R*^) *thi-1 hsdR17 supE44 relA1 lac* [F’ *proAB lacI^*q*^ZΔM15* Tn*10* (Tet^*R*^)]		NEB
BTH101	F^–^ *cya-99 araD139 galE15 galK16 rpsL1 (Str^*R*^) hsdR2 mcrA1 mcrB1*		Karimova et al., 1998; Ouellette et al., 2017
M15/pREP4	F^–^ *lac thi mtl*/pREP4		QIAGEN
BL21(DE3)	F^–^ *ompT gal dcm lon hsdS_*B*_(r_*B*_^–^ m_*B*_^–^)* (λDE3 *[lacI lacUV5-T7 gene 1 ind1 Sam7 nin5]*)		Studier and Moffatt, 1986
Plasmids		ori	
pSTM25	*aadA* P_*lac*_::*t25-TM*	p15A	Ouellette et al., 2014
pUTM18C	*bla* P_*lac*_::*t18-TM*	ColE1	Ouellette et al., 2014
pUTM18C-MalE	*malE* sequence (residues 27–396) cloned downstream T18-TM	ColE1	This study
pSTM25-LptA_*m*_	*lptA* sequence (residues 28–159) cloned downstream T25-TM	p15A	This study
pUTM18C-LptA_*m*_	*lptA* sequence (residues 28–159) cloned downstream T18-TM	ColE1	This study
pUTM18C-LptA_*m*_^*Q62L*^	*lptA* sequence (residues 28–159) bearing a Q-to-L mutation at position 62 cloned downstream T18-TM	ColE1	This study
pSTM25-LptA	*lptA* sequence (residues 28–185) cloned downstream T25-TM	p15A	This study
pSTM25-LptA^*Q62L*^	*lptA* sequence (residues 28–185) bearing a Q-to-L mutation at position 62 cloned downstream T25-TM	p15A	This study
pUTM18C-LptA	*lptA* sequence (residues 28–185) cloned downstream T18-TM	ColE1	This study
pUTM18C-LptA^*Q62L*^	*lptA* sequence (residues 28–185) bearing a Q-to-L mutation at position 62 cloned downstream T18-TM	ColE1	This study
pST25-LptC	*lptC* full-length sequence cloned downstream T25	p15A	This study
pUT18C-LptC	*lptC* full-length sequence cloned downstream T18	ColE1	This study
pQEsH-*lptC*	pQE30 (QIAGEN) derivative, expresses His_6_-LptC_24–191_; *bla*		Sperandeo et al., 2011
pET-LptAΔ_160–185_ –H	*pT7-lptAΔ_160–185_* -His_6_; *bla*		Laguri et al., 2017
			

### Plasmid Construction

To construct the recombinant plasmids used in the BACTH assay (listed in Table 1), the genes encoding the Lpt proteins of interest (or their subdomains) were PCR-amplified using the appropriate primer pairs, as listed in Table 2. The PCR products were then digested with the indicated restriction enzymes and subcloned into the corresponding sites of the pSTM25 and pUTM18C vectors. These BACTH vectors, expressing the T25 and T18 fragments of the adenylate cyclase toxin of *Bordetella pertussis* fused at their C-terminal ends with the first transmembrane domain of the *E. coli* OppB protein (TM), were employed in order to study protein interactions in the periplasm (Ouellette et al., 2014). In the recombinant plasmids pST25-LptC and pUT18C-LptC, full-length LptC (comprising its own transmembrane domain) was fused at the C-terminal end of the T25 and T18 fragments, respectively. MalE, LptA, and LptA_*m*_ were fused to the C-terminal end of TM to originate the constructs pUTM18C-MalE, pSTM25-LptA, pUTM18C-LptA, pSTM25-LptA_*m*_, and pUTM18C-LptA_*m*_. The recombinant plasmids pSTM25-LptA^*Q62L*^, pUTM18C-LptA^*Q62L*^, and pUTM18C-LptA_*m*_^*Q62L*^ were constructed by using a Q5 site-directed mutagenesis kit (New England Biolabs) with the primer pair AP733-AP734. Transformation was performed in XL1-Blue electrocompetent cells and transformants were selected at 30°C on LB plates supplemented with the appropriate antibiotics (ampicillin or spectinomycin), and 0.4% glucose to repress expression. All the cloned DNA regions obtained by PCR were verified by sequencing.

**TABLE 2:** Oligonucleotides.

Name	Sequence (5'–3')*^a^*		Used to make
AP576	Reverse	attgtggatccTTAAGGCTGAGTTTGTTTG	*lptC* cloning in pSTM25 and pUTM18C; BamHI
AP579	Forward	taatgtcgacgAAAATCGAAGAAGGTAAACTG	*malE* cloning in pUTM18C; SalI
AP580	Reverse	aaggatctagaTTACTTGGTGATACGAGTCTGC	*malE* cloning in pUTM18C; XbaI
AP581	Forward	gagacgagctcgGTAACCGGAGACACTGATCAG	*lptA* and *lptA*_*m*_ cloning in pUTM18C; SacI
AP582	Reverse	gagaggaattcTTAATTACCCTTCTTCTGTGC	*lptA* cloning in pUTM18C; EcoRI
AP665	Reverse	gagaggaattcTTAGCGCTTGCCTTTGTCG	*lptA*_*m*_ cloning in pUTM18C; EcoRI
AP666	Forward	aaggatctagagGTAACCGGAGACACTGATCAG	*lptA* cloning in pSTM25; XbaI
AP667	Reverse	attgtggatccTTAATTACCCTTCTTCTGTGC	*lptA* cloning in pSTM25; BamHI
AP688	Reverse	attgtggatccTTAGCGCTTGCCTTTGTCG	*lptA*_*m*_ cloning in pSTM25; BamHI
AP689	Forward	gaagatctgcagggATGAGTAAAGCCAGACGTTG	*lptC* cloning in pSTM25; PstI
AP690	Forward	gaagatctgcaggATGAGTAAAGCCAGACGTTG	*lptC* cloning in pUTM18C; PstI
AP733	Forward	ATCGTCACC**CTG**GGCACCATC	Q62L mutagenesis in *lptA*
AP734	Reverse	GACATTACCGGTAAAGGTAACC	Q62L mutagenesis in *lptA*

*^*a*^*E. coli* genomic sequence in uppercase; restriction sites in underlined lowercase; codon mutated by site-directed mutagenesis in bold.*

### Bacterial Adenylate Cyclase Two-Hybrid (BACTH) Assay

**FIGURE 2:**
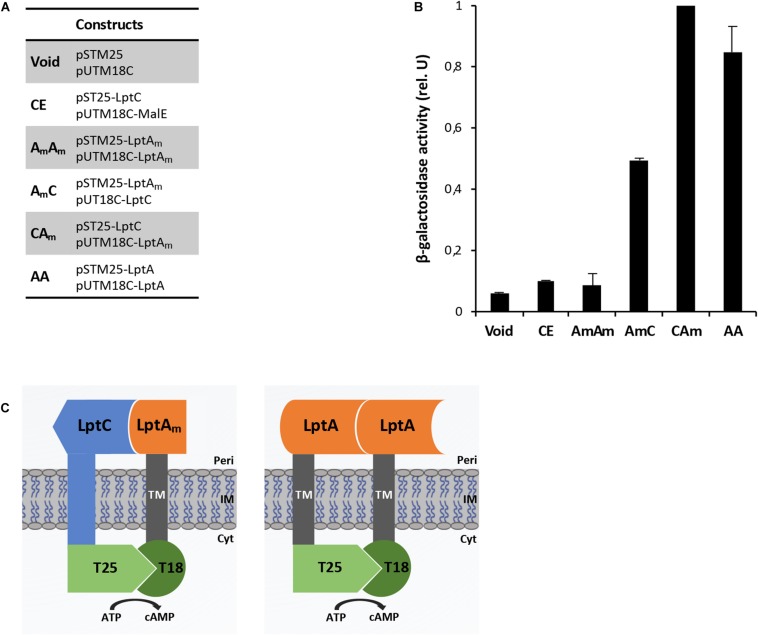
BACTH analysis of interactions between the tested combinations. **(A)** List of constructs co-expressed in the BTH101 strain for BACTH analysis. **(B)** β-Galactosidase activities are the results of at least two independent experiments in cells grown overnight in selective medium supplemented with IPTG. All values were normalized by the average activity obtained for T25LptC–TM18LptA_*m*_ (CA_*m*_) interaction. Each bar represents the mean value with standard deviation. **(C)** Schematic representation of the interactions T25LptC–TM18LptA_*m*_ (CA_*m*_) and TM25LptA–TM18LptA (AA). Full length LptC and truncated LptA were cloned in frame to the T25 and T18 fragments, respectively (left diagram). Full length LptA was fused in frame to both adenylate cyclase fragments (right diagram). TM indicates the transmembrane domain of OppB from *E. coli*. Peri, periplasm; IM, inner membrane; Cyt, cytoplasm.

To study protein–protein interactions with the BACTH system, electrocompetent BTH101 cells were co-transformed with each pair of plasmids to be tested (Figure 2A), plated onto LB plates containing selective antibiotics (100 μg/mL ampicillin and 50 μg/mL spectinomycin) and incubated at 30°C for 24–48 h. Interaction efficiencies were quantified by determining the β-galactosidase activities in 96-well microtiter plates according to a protocol adapted from Paschos et al. (2011). For this measurement, at least eight clones from each plasmid combination were analyzed for β-galactosidase activity in two independent experiments. Each clone was inoculated in 1 mL of LB medium supplemented with antibiotics and 0.5 mM IPTG for overnight induction. The β-galactosidase activity was measured from 20 μL culture diluted in 80 μL PM2 buffer (70 mM Na_2_HPO_4_. 12H_2_0, 30 mM NaH_2_PO_4_. H_2_O, 1 mM MgSO_4_, 0.2 mM MnSO_4_, pH 7.0) containing 8 mg/mL *ortho*-nitrophenyl-β-galactoside (ONPG), 0.01% SDS, and 50 mM β-mercaptoethanol. Reaction mixtures were incubated at room temperature for 20–30 min or until a sufficiently yellow color had developed, and the reactions were stopped with 100 μL 1 M Na_2_CO_3_. The optical densities at 420 and 550 nm were recorded for each sample using a plate reader (EnSpire Multimode Plate Reader, PerkinElmer) and the specific activity was calculated with the formula: Miller units = [OD_420_ - (1.75 × OD_550_)]/[*t* × OD_600_ × (volume in mL)] × 1000, where OD_600_ is the optical density at 600 nm after overnight incubation and *t* is the time in minutes needed for color formation.

### Peptide Synthesis

Peptides were synthesized on a 0.1 mmol scale on a Wang resin 0.99 mmol/g. The first amino acid was attached to the resin following a protocol described in the literature (Avitabile et al., 2019). The peptides were then elongated on a Liberty Blue CEM synthesizer using standard protocols. At the end of the synthesis, the peptides were cleaved from the resin and protecting groups were removed by treating the resin with a solution of TFA/thioanisol/H_2_O 95/2.5/2.5 v/v/v for 2 h. The peptides were then lyophilized. Wild type (WT) peptide was cyclized as reported by Fehlbaum et al. (1996). Peptides were purified by RP-HPLC on a Jupiter 10μ Proteo 90A° (100 × 21.20 mm) column using a gradient of CH_3_CN (0.1% TFA) in H_2_O (0.1% TFA) from 10 to 50% in 20 min and analyzed on a Vydamass C18 100A 5μ 150 × 4.6 mm column with the same gradient. Peptides were characterized by mass spectrometry on a Thermo Scientific LCQ Fleet ion trap. Pure peptides were then lyophilized three times, the first to eliminate HPLC solvents, the second from a solution 6/4 v/v H_2_O/CH_3_COOH, and the third in water.

#### Thanatin WT Cyclic

Sequence: GSKKPVPIIYCNRRTGKCQRM
Calculated mass (Da): 2433.95; found (Da): 1217.08 [M+2H]^2+^; 812.33 [M+3H]^3+^; 609.29 [M+4H]^4+^

#### Thanatin Scramble (Scr)

Sequence: YVCIRMNKISPKQRTPGGRCK
Calculated mass (Da): 2435.95; found (Da): 1219.02 [M+2H]^2+^; 813.47 [M+3H]^3+^; 610.50 [M+4H]^4+^

### Determination of Minimal Inhibitory Concentration (MIC)

The minimal inhibitory concentration (MIC) values of thanatin, thanatin scramble, and vancomycin (as a positive control) were assessed with a protocol adapted from Wiegand et al. (2008) using 96-well microtiter plates. Stationary phase cultures of the *E. coli* WT strain MG1655 (Blattner et al., 1997), the permeabilized mutants AS19 (Sekiguchi and Iida, 1967) and NR698 (Ruiz et al., 2005), and the BACTH strain BTH101 (Karimova et al., 1998; Ouellette et al., 2017) grown at 37°C in LB medium, were diluted in fresh medium adjusting the OD_600_ to a value of 0.05 and incubated in the presence of twofold decreasing concentrations of the compounds ranging from 64 μg/mL to 62.5 ng/mL. After 24 h of incubation at 37°C, the OD_600_ was measured by a plate reader (EnSpire Multimode Plate Reader, PerkinElmer). The MIC value was determined as the lowest concentration of compound leading to no detectable growth.

### Analysis of Thanatin’s Effect on Lpt Protein Interactions Using the BACTH Assay

To assess thanatin’s effect on the periplasmic interactions LptA–LptA and LptC–LptA_*m*_, at least four clones from each combination were cultured in LB medium supplemented with antibiotics at 37°C to an OD_600_ around 1.0. These precultures were used to inoculate 1 mL of LB medium supplemented with antibiotics, 0.5 mM IPTG, and thanatin at different concentrations to an OD_600_ of 0.05; and the cultures were incubated for 18 h (overnight) at 30°C. After overnight induction of the expression of the hybrid proteins, the β-galactosidase activities were determined. For the clones expressing the BACTH combination T25LptC-TM18LptA_*m*_, thanatin was tested at 0.7, 1.0, and 1.4 μg/mL. For the TM25LptA–TM18LptA pair, a higher concentration of thanatin could be added to the cultures without affecting bacterial growth; thus, values of 0.7, 1.0, 1.4, and 2.8 μg/mL were tested. A scrambled version of thanatin (Scr) was also employed in this assay at the same concentrations as a control for the specificity of interaction inhibition.

### Protein Production and Purification

*Escherichia coli* LptC lacking the first 23 residues of the transmembrane domain was expressed from a plasmid (LptC pQESH, QIAGEN) with an N-terminal His-Tag and purified as described (Laguri et al., 2017). LptC was expressed in ^15^N enriched deuterated medium with specific ^13^C-^1^H labeling of Isoleucines δ1 and Leucine and Valine proR methyl groups according to standard protocols (Kerfah et al., 2015) with NMRbio precursors^1^. LptA_*m*_ coding for residues 28–159 followed by a SGRVEHHHHHH TAG in a pET21b vector was expressed and purified as described (Laguri et al., 2017). Both proteins were exchanged to 50 mM Na_2_HPO_4_ pH 8.0, 150 mM NaCl buffer.

### NMR Spectroscopy

Nuclear magnetic resonance experiments were recorded at 25°C on Bruker 600 MHz spectrometer equipped with a triple resonance cryoprobe. 2D-[^1^H, ^13^C]-methyl-SOFAST experiments were recorded to follow LptC methyl groups on LptC ^15^N^2^H and ^13^C-^1^H specifically labeled on Iδ1, Lδ1, and Vγ1 at 20 μM prepared in 50 mM Na_2_HPO_4_ pH 8.0, 150 mM NaCl buffer with 10% D_2_O. Unlabeled LptA_*m*_ at 40 μM prepared in the exact same buffer was added to LptC to achieve 100% of LptC complexed with LptA_*m*_. Thanatin or Scr at 42 μM was added to the complex and interaction experiments were followed using 2D-[^1^H, ^13^C]-methyl-SOFAST experiments. NMR experiments were processed and analyzed using Topspin 3.2 and CcpNmr 2.4.

### Biacore Experiments

Surface plasmon resonance (SPR) experiments were performed on a Biacore T200 with a CM3 chip. HBS-P+ and HBS-N buffers (GE Healthcare) were used for immobilization and interactions, respectively. 66 Resonance units (RUs) of LptA_*m*_ were immobilized on a flow cell by the amine (EDC-NHS) coupling method followed by ethanolamine saturation, with a flow cell modified only with EDC-NHS-ethanolamine as reference for subtractions. For interactions, protein and ligands were diluted in HBS-N running buffer and regeneration between injections achieved with a 30 s pulse of 10 mM HCl. Sensorgrams shown were subtracted with the reference flow cell as well as with injection of buffer alone. Determination of LptC–LptA_*m*_ Kd was performed by injecting increasing concentrations of LptC (5.6–100 μM) over immobilized LptA_*m*_. Kinetics analysis of the data was unsuccessful due to very fast association, and the Kd was determined from steady-state binding levels obtained at the end of the association phase with Bioeval software (GE Healthcare).

### Determination of LptA, LptD, and LptB Steady-State Levels Upon Thanatin Treatment

LptA, LptD, and LptB (as loading control) steady-state levels were assessed in the MG1655 strain by western blot analysis with polyclonal antibodies raised in rabbit against LptA, LptD, and LptB. Bacterial cultures were grown at 37°C in LB medium. At OD_600_ 0.1, the cells were treated or not with 5.25 μg/mL of thanatin (1.5 × MIC). Cell growth was monitored by measuring the OD_600_ value at 30-min intervals and viability was determined by quantifying the colony-forming units (CFU) at 1-h intervals during a time period of 4 h. Whole-cell extracts for protein analysis were collected and harvested by centrifugation (5000 *g*, 10 min) 20, 30, 40, 60, and 120 min after treatment with thanatin. The cell pellets were resuspended in a volume (in mL) of SDS Laemmli buffer equal to 1/24 of the total optical density of the sample. The samples were boiled for 5 min and equal volumes (15 μL) were separated by 12.5% SDS-PAGE. Proteins were transferred onto nitrocellulose membranes (GE Healthcare), and immunodecoration was performed as previously described (Sperandeo et al., 2007). Polyclonal antibodies raised against LptA (GenScript Corporation), LptD (GenScript Corporation), and LptB (kindly provided by D. Kahne and N. Ruiz) were used as primary antibodies at dilutions of 1:1,000, 1:500, and 1:10,000, respectively. As secondary antibody, goat anti-rabbit immunoglobulin (Li-Cor) was used at a dilution of 1:15,000. Bands were visualized by an Odyssey Fc imaging system (Li-Cor GmbH).

### LPS Analysis From Whole-Cell Extracts

Whole-cell extract samples for LPS analysis were obtained as described in the previous section. For LPS visualization, equal volumes (20 μL) of whole-cell extracts were digested with 6 μg of proteinase K (Sigma–Aldrich) at 60°C for 1 h and then separated by 18% Tricine SDS-PAGE (Lesse et al., 1990). Immunodecoration was performed using anti-LPS core WN1 222-5 monoclonal antibodies (Hycult Biotech) at a dilution of 1:500. As secondary antibody, goat anti-mouse immunoglobulin G-peroxidase (HRP) conjugate (Sigma–Aldrich) was used at a dilution of 1:5000.

## Results

### Adaptation of the BACTH Assay for the Detection of Lpt Protein Interactions in the Periplasm

The BACTH system was implemented in this work to allow the detection *in vivo* of two crucial protein-protein interactions within the Lpt interactome, namely, LptC–LptA and LptA–LptA. The BACTH assay is based on the interaction-mediated reconstitution of the adenylate cyclase activity of the toxin of *B. pertussis*, whose catalytic domain can be divided in two complementary fragments, T25 and T18 (Karimova et al., 1998; Battesti and Bouveret, 2012). In this work, we used the BACTH vectors expressing these fragments fused in frame with the first transmembrane domain of the *E. coli* OppB protein (TM) (pSTM25 and pUTM18C). These plasmids allow expression of the targeted protein domains fused to TM25 and TM18 into the periplasm (Ouellette et al., 2014) which reflects the physiological environment of the tested interactions. To detect LptA–LptA dimerization, LptA was subcloned into both BACTH vectors at the C-terminal end of the TM, originating the hybrid TM25LptA and TM18LptA proteins. To detect LptC–LptA association, we fused at the C-terminal end of the TM a truncated monomeric version of LptA, referred to as LptA_*m*_, that lacks the last C-terminal β-strand and is not able to self-oligomerize, although still functional *in vivo* (Laguri et al., 2017). We decided to use LptA_*m*_ to avoid titration of the fusion protein caused by interaction of LptA with itself, leading to a decrease in the β-galactosidase signal when testing LptC–LptA interaction with the BACTH technique. Full-length LptC was subcloned into both pSTM25 and pUTM18C vectors, in frame with the C-terminal end of the adenylate cyclase fragments, to obtain the constructs T25LptC and T18LptC.

As negative controls for the assay, we used: (i) the combination between the void plasmids pSTM25 and pUTM18C; (ii) the non-productive LptA_*m*_–LptA_*m*_ association; and (iii) the association LptC–MalE, between LptC and the unrelated periplasmic binding subunit of the *E. coli* maltose transporter, MalE (Davidson et al., 1992; Ehrmann et al., 1998). Constructs were transformed into the adenylate cyclase-deficient strain BTH101 and the efficiency of interaction between the various protein fusions was quantified by measuring the β-galactosidase activity. The results for the BACTH complementation assay are presented in Figures 2A,B. As expected, LptC–MalE combination did not produce a positive interaction signal, confirming that the BACTH system is suitable to detect specific interactions occurring in the periplasm. Also, truncated LptA was confirmed to be unable to oligomerize. We successfully detected *in vivo* the LptC–LptA_*m*_ interaction and the dimerization of LptA (schematic representation in Figure 2C). The signal obtained for the pair T25LptC–TM18LptA_*m*_ (CA_*m*_) was twofold higher than the one obtained for the complementary combination TM25LptA_*m*_–T18LptC (A_*m*_C). This effect is not surprising since it was previously reported that β-galactosidase measurements may significantly vary according to the T25 and T18 combination chosen for the BACTH assay (Ouellette et al., 2017). Indeed, when testing the LptC–LptA_*m*_ interaction, hybrid LptA_*m*_ can be titrated away from the reaction by interaction through its N-terminal with native LptA. This effect is likely even more significant in the TM25LptA_*m*_–T18LptC configuration, where LptA_*m*_ is expressed from a low-copy number vector (pSTM25), thus further diminishing the number of hybrid LptA_*m*_ proteins free to interact with LptC and accounting for the lower β-galactosidase signal observed in A_*m*_C combination. Therefore, we decided to use the pair of constructs T25LptC–TM18LptA_*m*_ in further tests. It should be noted that in our assay, the interaction of full-length LptA with itself (LptA–LptA) produced a lower β-galactosidase activity signal compared to LptC–LptA_*m*_. This is consistent with previously published *in vitro* measurements revealing that the affinity between LptA and LptC is stronger than the affinity for LptA oligomerization (Schultz et al., 2013).

### Thanatin Inhibits LptC–LptA_*m*_ and LptA–LptA Interactions *in vivo*

**FIGURE 3:**
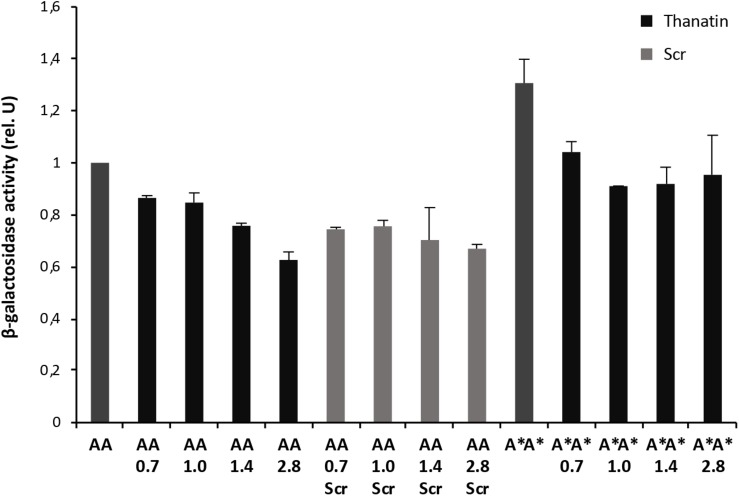
Thanatin’s effect on LptA–LptA (AA) and LptA^*Q62L*^–LptA^*Q62L*^ (A*A*) interactions. Thanatin and thanatin scramble (Scr) were tested at concentrations ranging from 0.7 to 2.8 μg/mL. All values were normalized by the average activity obtained for the untreated LptA–LptA interaction. Values are averages of at least eight technical replicates from two independent experiments ± standard deviation.

**FIGURE 4:**
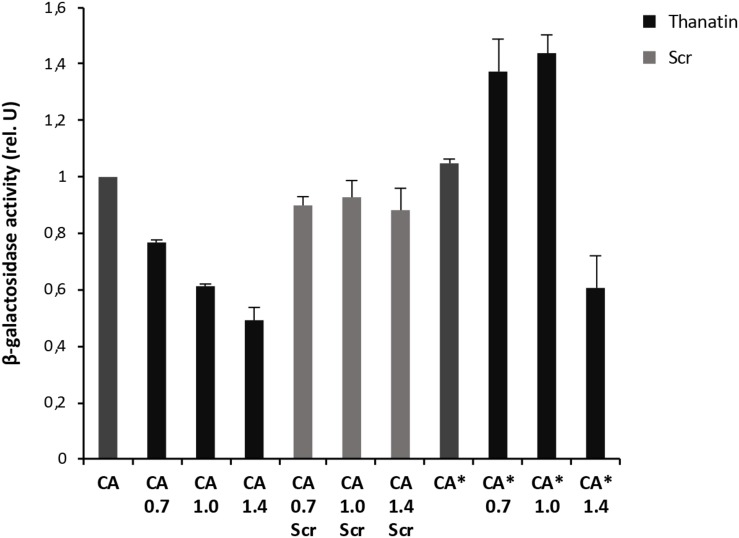
Thanatin’s effect on LptC–LptA_*m*_ (CA) and LptC–LptA_*m*_^*Q62L*^ (CA*) interactions. Thanatin and thanatin scramble (Scr) were tested at concentrations ranging from 0.7 to 1.4 μg/mL. All values were normalized by the average activity obtained for the untreated interaction LptC–LptA_*m*_. Values are averages of at least eight technical replicates from two independent experiments ± standard deviation.

Interaction between the antibacterial peptide thanatin and LptA was recently demonstrated and NMR experiments clearly showed that the N-terminal strand of the β-hairpin of thanatin docks in parallel orientation onto the first N-terminal β-strand of the β-jellyroll of LptA (Vetterli et al., 2018). It is well known from structural studies that dimerization of LptA monomers with themselves or with LptC involves the N-terminal edge strand of the β-jellyroll of LptA (Suits et al., 2008; Schultz et al., 2013; Laguri et al., 2017). Thanatin’s binding site therefore overlaps with the interaction site of LptA with another LptA subunit in the homodimer LptA–LptA and with LptC in the heterodimer LptA–LptC, suggesting a possible mechanism for the antibacterial activity. We thus analyzed the effect of thanatin on these interactions with the adapted BACTH assay and used a scrambled version of thanatin, characterized by the same amino acid composition but with a different sequence (thanatin scramble, Scr), as specificity control (Figures 3, 4). The MIC of thanatin and thanatin scramble was assessed against WT MG1655, and permeabilized AS19 and NR698 *E. coli* strains. The MIC values were 1.8–3.5 and above 64 μg/mL for thanatin and thanatin scramble, respectively, when tested against the WT MG1655 strain (Table 3). Slightly lower MIC values were obtained for thanatin when tested against the permeabilized *E. coli* mutants (0.1–0.4 μg/mL). On the contrary, no significant difference relative to the WT strain was observed in the MIC values of thanatin scramble when tested against the permeabilized mutant strains, suggesting that the lower activity of the peptide cannot be attributed to its inability to cross the OM barrier.

**TABLE 3:** Minimal inhibitory concentrations (MICs) in μg/mL of thanatin, thanatin scramble, and vancomycin.

	MIC (μg/mL)		
Strains	Thanatin	Thanatin scramble	Vancomycin
MG1655	1.8–3.5	>64	>64
AS19	0.1–0.2	64	4.0–8.0
NR698	0.1–0.4	32–64	0.5–1.0
BTH101	3.5	>64	>64

*Compounds were tested against wild-type (MG1655) and permeable (AS19 and NR698) *Escherichia coli* strains. The BTH101 strain used in the BACTH assay was also tested. The data are representative of three biological replicates.*

To explore whether thanatin’s antibacterial activity is due to the inhibition of LptA interaction with itself or with LptC, we evaluated the effect of increasing sub-MIC concentrations of the peptide on the TM25LptA–TM18LptA and T25LptC–TM18LptA_*m*_ associations and the results are presented in Figures 3, 4, respectively. After overnight induction of the fusion proteins in the presence of thanatin, we could observe inhibition not only of LptA–LptA dimerization but also of LptC–LptA_*m*_ interaction, but the inhibitory effect was much greater on the latter. A clear dose-dependent response could only be observed in the inhibition of LptC–LptA_*m*_ interaction. The thanatin scramble was not capable of disrupting these periplasmic interactions in a dose-dependent manner, indicating that this is an effect specific to thanatin secondary and tertiary structures rather than to any cationic peptide with the same amino acid composition.

We also tested a previously isolated thanatin-resistant mutant presenting a glutamine to leucine substitution at position 62 in the LptA protein (*lptA*^*Q62L*^ allele) (Vetterli et al., 2018). The *lptA-Q62L* mutation was introduced into the BACTH constructs and tested as described above (Figures 3, 4). The data obtained suggest that Q62L mutation in LptA specifically impairs the ability of thanatin to disrupt LptC–LptA_*m*_^*Q62L*^ (CA^∗^) association, since the peptide is not effective against CA^∗^ at concentrations at which it is active against the WT CA pair, namely, 0.7 and 1 μg/mL (Figure 4). It should be noted that Q62L mutation exerts an unexpected stabilizing effect on LptA^*Q62L*^–LptA^*Q62L*^ (A^∗^A^∗^) interaction, resulting in a β-galactosidase signal higher than that of the WT LptA–LptA combination. This effect is abolished upon treatment with thanatin, although not in a dose-dependent manner (Figure 3). Residue Q62 is not directly involved in the interaction of LptA with another LptA monomer, LptC, or with thanatin but belongs to a loop of the β-jellyroll of LptA that comes into contact with the short N-terminal α-helix of the WT protein upon thanatin interaction (Vetterli et al., 2018). This effect could be explained assuming that Q62L mutation induces a conformational change in the N-terminal region of LptA, which alters the way all these three interactions occur. Since thanatin’s binding site overlaps the binding site of LptA with another LptA protein, if the Q62L mutation somehow alters thanatin’s binding, then it is possible that it also alters the interaction of LptA^*Q62L*^ with itself, perhaps by strengthening it.

A similar stabilizing effect is also observed when testing the LptC–LptA_*m*_^*Q62L*^ combination. However, in this case, the effect is observed only upon treatment with low concentrations of thanatin, since the β-galactosidase signal of non-treated CA^∗^ is comparable to that of the WT (Figure 4). This suggests that LptA_*m*_^*Q62L*^ is still able to bind thanatin and this binding determines a conformational change in the protein that somehow enhances the stability of LptC–LptA_*m*_^*Q62L*^ complex. At higher thanatin concentrations, however, it seems that the inhibitory effect of the peptide on LptC–LptA_*m*_^*Q62L*^ prevails over the stabilizing effect. This could be explained by hypothesizing that Q62L mutation in LptA creates a secondary high-affinity binding site for thanatin. According to this hypothesis, when low concentrations of thanatin are added, thanatin binds to the high-affinity site, leaving the binding site for LptC unoccupied (and possibly stabilizing LptC–LptA_*m*_^*Q62L*^ complex). On the contrary, when higher concentrations of thanatin are used, all the available binding sites are occupied, thus impairing LptC–LptA complex formation.

### Thanatin Disrupts LptC–LptA_*m*_ Interaction *in vitro*

**FIGURE 5:**
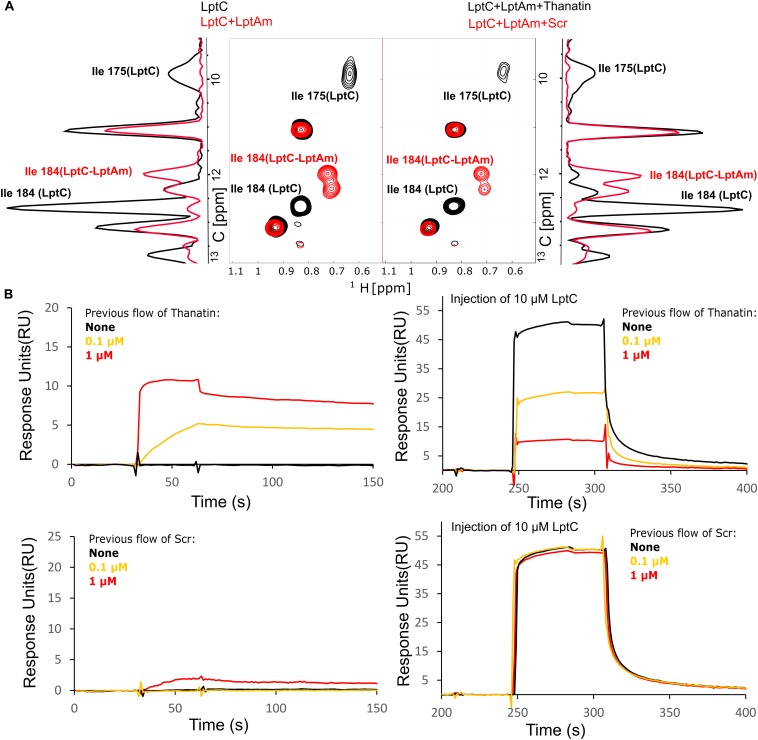
LptC–LptA interaction is specifically disrupted by thanatin. **(A)** [^1^H, ^13^C]-correlation spectra of methyl labeled LptC in absence (black) and presence (red) of two molar equivalents of LptA_*m*_, focused on Isoleucines in black for signals corresponding to isolated LptC and in red for LptC in complex with LptA_*m*_ (left panel). A 1D-^13^C projection of the 2D experiment is shown on the side for clarity. On the right panel, addition of thanatin disrupts LptC–LptA_*m*_ interaction originating signals characteristic of LptC alone (black). Thanatin scramble (Scr) did not change LptC–LptA_*m*_ spectra, indicating intact LptC–LptA_*m*_ complexes (red). **(B)** Monitoring of LptC–LptA_*m*_ interaction upon thanatin treatment by SPR. (Left panels) Thanatin or Scr were injected over surface-immobilized LptA_*m*_, with concentrations following a color-code in bold: 0 (black), 0.1 μM (yellow), or 1 μM (red). Thanatin interacts strongly with LptA_*m*_ presenting rapid association and slow dissociation. (Right panels) For clarity, after previous injections with the same concentrations of thanatin/Scr, sensorgrams were readjusted to 0, followed by LptC injection at 10 μM and recording. Binding of LptC to immobilized LptA_*m*_ is reduced with increasing thanatin concentrations previously flowed (maximum at 1 μM), while Scr displays no effect.

Thanatin’s ability to interfere with LptC–LptA complex formation was assessed by NMR and SPR. For these assays, the monomeric version of LptA (LptA_*m*_) was used to neglect the oligomerization of LptA. ^1^H-^13^C NMR of the specifically labeled Isoleucines of LptC efficiently report on the interaction with LptA (Laguri et al., 2017). In particular, Isoleucines 175 (175Ile) and 184 (184Ile) δ1 methyl groups at the C-terminus of LptC and in the vicinity of the binding interface change chemical shifts upon formation of the complex with LptA_*m*_ (Figure 5A, left panel). After adding thanatin to the LptC–LptA_*m*_ complex, we observed that 175Ile and 184Ile peaks completely shifted to a frequency corresponding to free LptC, indicating a total disruption of LptC–LptA_*m*_ dimers (Figure 5A, right panel). The same experiment performed with the thanatin scramble (Scr) showed no disruption of the LptC–LptA_*m*_ complex, suggesting specific competition and disruption of the binding interface by the thanatin (Figure 5A, right panel).

Complex disruption was also probed by SPR, in which the surface of a chip was functionalized with LptA_*m*_. First, we confirmed the binding of LptC to the immobilized LptA_*m*_ (Supplementary Figure S1A) and we determined the Kd of the interaction (Kd = 80 ± 44 μM). Then, to assess thanatin’s effect, we injected thanatin over LptA_*m*_ and confirmed stable interaction with LptA_*m*_ on the surface, followed by the injection of LptC (Figure 5B, upper panels). We observed that, upon LptC injection, the response values decreased in a dose-dependent manner to the thanatin injected in the system (in concentrations up to 1 μM), indicating fewer surface-free LptA_*m*_ epitopes available to interact with LptC (Figure 5B, upper right panel). The same experiment with the scrambled version showed no or little binding of Scr to immobilized LptA_*m*_ and hence no effect on LptC binding (Figure 5B, lower panels), further demonstrating a specific effect of thanatin in preventing the formation of LptC–LptA_*m*_ complex.

### Thanatin Treatment Results in LptA Degradation and LPS Modification

**FIGURE 6:**
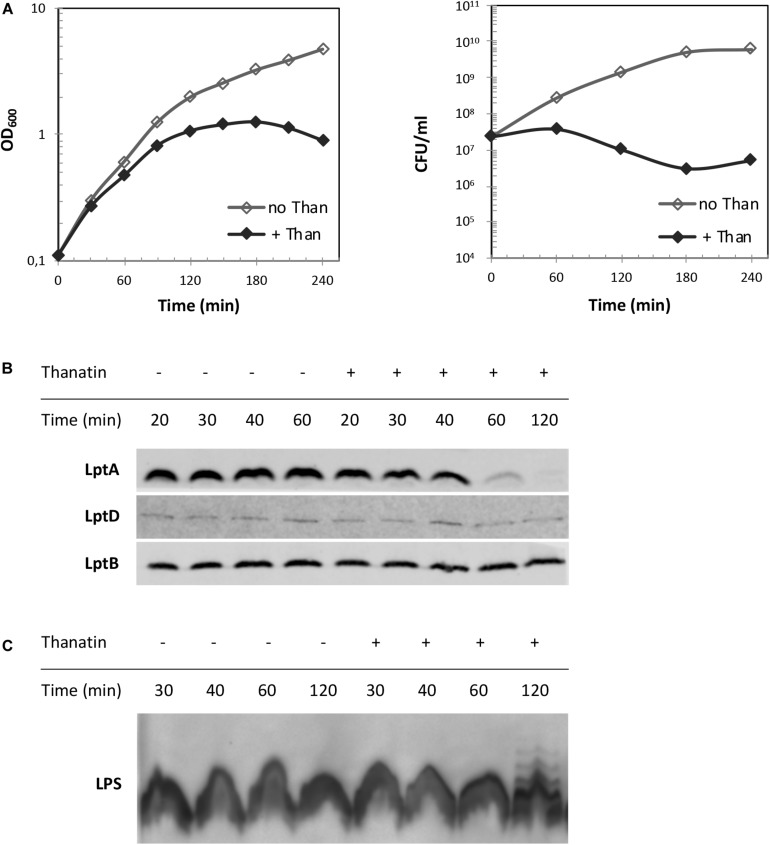
LptA steady-state level and LPS profile upon treatment with thanatin. **(A)** Growth curves of wild type strain MG1655 in the presence (+ Than) and absence (no Than) of thanatin at 5.25 μg/mL (1.5 × MIC) concentration. Growth was monitored by measuring the OD_600_ (left panel) and by determining CFU/mL (right panel). **(B)** Western blot analysis to reveal LptA steady-state levels. Samples were collected 20, 30, 40, 60, and 120 min after treatment with thanatin. Whole-cell extracts were prepared and analyzed by western blot with anti-LptA, anti-LptD, anti-LptB (as loading control) antibodies. An equal amount of cells (0.36 OD_600_ units) was loaded into each lane. **(C)** Western blot analysis to reveal LPS profiles. Whole-cell extracts obtained 30, 40, 60, and 120 min after treatment with thanatin were incubated with proteinase K and analyzed by western blot with anti-LPS antibodies. An equal amount of cells (0.48 OD_600_) was loaded into each lane. Results shown are representative of three independent experiments.

Depletion of components of the IM and OM Lpt sub-complexes results in LptA degradation, which has been proposed to be a marker of incorrect complex assembly (Sperandeo et al., 2011). We reasoned that the disruption of LptC–LptA interaction by thanatin treatment could impair Lpt complex assembly. Therefore, we evaluated the LptA steady-state levels in *E. coli* WT cells upon treatment with thanatin. Samples were taken at different time points within 2 h from MG1655 cultures grown in the presence or absence of thanatin at 5.25 μg/mL (1.5 × MIC) and analyzed by western blotting using anti-LptA antibodies. The abundance of LptD, the OM docking element of LptA, was also assessed and the level of LptB was used as a sample loading control. Culture growth and cell viability were monitored by OD_600_ measurement and determination of CFU, respectively, for a time span of 4 h. In cultures treated with thanatin, we observed a decrease in the OD_600_ with minor effect on cell viability (Figure 6A). As shown in Figure 6B, substantial LptA degradation occurs within 60 min of incubation with thanatin and, after 120 min, the steady-state level of LptA is very low and almost undetectable with our antibody preparation. The abundance of LptD did not change over time, indicating that the steady-state level of this OM component is not affected by thanatin treatment. The decrease in LptA level suggests that the IM and OM are not properly bridged when cells are treated with thanatin.

Depletion of any Lpt component leads to the accumulation of LPS decorated with colanic acid repeating units at the IM outer leaflet (Ruiz et al., 2008; Sperandeo et al., 2008). This phenotype is diagnostic of defects in Lpt occurring after MsbA-mediated flipping of lipid A-core across the IM. We therefore tested whether treatment with thanatin would induce similar LPS modifications. As shown in Figure 6C, LPS decorated with colanic acid, migrating as ladder-like bands in gel electrophoresis, was detected 120 min after adding thanatin to the culture; no LPS modification was observed in untreated cells. These data suggest that thanatin, by disrupting the LptC–LptA interaction, impairs Lpt complex assembly leading to the accumulation of LPS at the periplasmic side of the IM, where it is decorated with colanic acid. The observed LPS profile, together with the LptA degradation kinetic, strongly suggests that the disruption of the Lpt protein bridge could be the major killing mechanism of thanatin against Gram-negative bacteria.

## Discussion

The LPS export pathway is a valuable target for novel antibiotic discovery. Murepavadin, a macrocyclic peptidomimetic, has thus far been the most promising antibiotic candidate targeting the Lpt machinery. It was originally identified from a library of structural mimics of class I CAMP (cationic antimicrobial peptide) protegrin and later found to target the β-barrel OM protein LptD (Srinivas et al., 2010; Werneburg et al., 2012; Andolina et al., 2018).

Recent efforts to target the Lpt pathway have led to the identification, through a YTH assay, of IMB-881 as a synthetic molecule inhibiting LptC–LptA interaction (Zhang et al., 2019). The inhibitory activity of IMB-881 further suggests that interfering with the Lpt interactome is a good strategy to prevent Lpt to the cell surface. Nevertheless, in the YTH system, LptC–LptA interaction occurs in the cytoplasm of a yeast cell, and molecules active in this system may not be able to permeate the bacterial OM. To improve the screening system, we here implemented the BACTH assay (Karimova et al., 1998; Ouellette et al., 2014, 2017) that enables targeting of Lpt protein interactions in their native environment, preserving both protein functionality and folding state. This bacterial two-hybrid technique was used to probe LpC–LptA and LptA–LptA interactions. LptC has an important structural role in the Lpt machinery as it serves as the docking site for LptA binding to the IM LptB_2_FGC complex (Sperandeo et al., 2011; Freinkman et al., 2012). Indeed, mutations in LptC compromising interaction with LptA are lethal (Sperandeo et al., 2011; Villa et al., 2013). LptA molecules have a strong tendency to oligomerize in solution (Suits et al., 2008; Merten et al., 2012; Santambrogio et al., 2013) but we still do not know whether LptA self-oligomerization has a physiological relevance, since a monomeric LptA is still able to partially support cell growth (Laguri et al., 2017). In the BACTH assay, LptC–LptA interaction appears stronger than LptA–LptA dimerization, in line with the reported *in vitro* affinities (Schultz et al., 2013); however, we cannot exclude that the observed lower β-galactosidase signal could also be due to the formation of non-productive interactions between LptA molecules fused to the same (T25 or T18) adenylate cyclase fragment. The assay seems robust as no association is detected between unrelated non-interacting proteins: LptC and the maltose periplasmic binding protein MalE (Davidson et al., 1992) or between oligomerization deficient LptA_*m*_ proteins (Laguri et al., 2017).

The BACTH system was also employed to explore the mechanism of action of thanatin, an antimicrobial peptide recently shown to bind the first β-strand of LptA (Vetterli et al., 2018). Thanatin inhibits LptC–LptA interaction in a dose-dependent manner, whereas very little and non-dose dependent inhibitory effect is observed against LptA–LptA association. LptA first N-terminal β-strand is a key determinant interacting with the C-terminal region of LptC or the C-terminal region of another LptA in head-to-tail LptA self-oligomerization (Freinkman et al., 2012; Laguri et al., 2017). LptC and LptA share a very similar protein architecture, despite no amino acid sequence similarity (Tran et al., 2010; Villa et al., 2013); indeed, in the LptC–LptA_*m*_ complex, LptC precisely occupies the same position as LptA in the LptA oligomer (Laguri et al., 2017). Interestingly, thanatin seems able to discriminate between the two different interactions that LptA is implicated on via its N-terminal region and could, therefore, also serve as a tool to probe the different interactions occurring within the Lpt periplasmic protein bridge. This result is in agreement with earlier data showing that the enantiomeric form of thanatin (D-thanatin) is nearly inactive against Gram-negative strains, suggesting that a stereospecific recognition by a cellular target is required for thanatin to exert its antibacterial effect (Fehlbaum et al., 1996).

A scrambled version of thanatin, that maintains the overall peptide amino acid composition and charge, loses the antibacterial activity, fails to disrupt the LptC–LptA interaction *in vivo* (BACTH assay) and *in vitro* (NMR), and does not bind to LptA_*m*_ (SPR analyses). These data further support a specific action of thanatin in binding to LptA and in competing with LptC for the formation of the LptC–LptA complex. Thanatin scramble does not display antibacterial activity against permeabilized *E. coli* strains, strongly suggesting that the lack of activity of the scrambled peptide is not due to its inability to reach its target in the periplasm.

It has been reported that *E. coli* cells carrying LptA^*Q62L*^ amino acid substitution become resistant to thanatin (Vetterli et al., 2018). Residue Q62 does not appear to be implicated in thanatin binding and the mechanism underlying resistance is still unknown. LptC–LptA^*Q62L*^ interaction is not inhibited by thanatin in the BACTH assay and, surprisingly, it appears stronger in the presence of the peptide. In the case of the interaction between LptA^*Q62L*^ mutant proteins, the dimerization seems stronger than that observed between WT LptA, even in the absence of thanatin. We can speculate that Q62L mutation somehow alters the stability of both LptC–LptA and LptA–LptA complexes, affecting the binding of thanatin to LptA. However, it is difficult to explain these results since neither the effect of the Q62L substitution on LptA structure nor the mechanism of thanatin resistance are known.

Previous *in vitro* data revealed that besides LptA, thanatin binds to the LptDE complex in the low nanomolar range and, furthermore, its binding site in LptA has been shown by modeling studies to be highly conserved in the periplasmic domain of LptD (Robinson, 2019). This suggests that thanatin can inhibit multiple protein–protein interactions required for the Lpt complex assembly. It was not possible to test the periplasmic domain of LptD in the BACTH assay, since expression of a folded and functional LptD is strictly dependent on the expression and interaction with LptE (Chng et al., 2010). Nevertheless, the isolation of suppressor mutants exclusively at the N-terminal region of LptA (Vetterli et al., 2018), that is not involved in the LptA–LptD interaction, and the ability of the LptA_*m*_ mutant protein, lacking the C-terminal β-strand implicated in both LptA–LptA and LptA–LptD interactions, to partially support the cell growth (Laguri et al., 2017) suggest that LptC–LptA interaction is thanatin’s main target.

Thanatin has been related to the group of CAMPs that kill bacteria by cell agglutination. In the host organism, this class of antimicrobial peptides does not permeabilize bacterial cell membranes but rather interacts with LPS or peptidoglycan, favoring cell aggregation and bacterial removal by phagocytosis (Shai, 2002; Jung et al., 2012; Pulido et al., 2012). Thanatin has indeed been shown to bind LPS *in vitro* and promote cell agglutination as a result of cell surface charge neutralization (Sinha et al., 2017). Recently, the comparison of thanatin’s affinity to LPS relative to Ca^2+^ and Mg^2+^ revealed that thanatin displaces divalent cations from LPS *in vivo* promoting LPS shedding from bacterial cells at concentrations 10-fold higher than the MIC, increasing OM permeability (Ma et al., 2019). Interestingly, the same study reports that thanatin is able to inhibit the enzymatic activity of New Delhi metallo-β-lactamase-1 (NMD-1), responsible for the resistance to β-lactam antibiotics in several multidrug resistant strains, by binding to the active site of the enzyme with higher affinity than Zn^2+^, displacing it and reversing carbapenem resistance. This evidences that, alongside a killing effect on Gram-negative pathogens based on OM permeabilization, thanatin may help restoring the activity of β-lactam antibiotics in multidrug resistant pathogens (Ma et al., 2019).

In the reported BACTH assay, inhibition of LptC–LptA interaction is observed at sub-MIC concentrations of thanatin, a condition that does not inhibit the growth of cells expressing LptC and LptA_*m*_ protein fusions. Based on our data, we propose that LPS binding is employed by thanatin as a self-promoted mechanism of entry in the periplasm of bacterial cells where the LptA target resides. Supporting this hypothesis is the finding of a mutated version of thanatin, where Arg 13 and Arg 14 residues have been substituted by Ala, that presents reduced LPS binding affinity and loses the antibacterial activity (Sinha et al., 2017).

In *E. coli* cells treated with thanatin, LptA undergoes degradation and LPS is decorated with colanic acid. Notably, these phenotypes are observed in cells where LPS export machinery disassembles and transport of LPS molecules is impaired due to mutations in any of the Lpt complex components (Sperandeo et al., 2008, 2011). These data suggest that the main mechanism of action of thanatin occurring at MIC concentration is the disassembly of the Lpt machinery and consequently the blocking of LPS transport.

Overall, our results highlight OM biogenesis as an excellent target for novel antibiotic discovery. Thanatin joins the increasing list of molecules that disrupt the assembly of the OM with diverse mechanisms (Hart et al., 2019; Imai et al., 2019; Lehman and Grabowicz, 2019; Psonis et al., 2019). Based on their mechanisms, these compounds could be employed not only to fight multidrug resistant pathogens but also in combination with existing antibiotics not sufficiently effective.

## Data Availability Statement

All datasets generated for this study are included in the article/Supplementary Material.

## Author Contributions

EM performed the BACTH assays and *in vivo* experiments. TB and CL performed NMR and SPR experiments. AR and EE designed and synthetized the peptides. AP, PS, AM, and EM designed the *in vivo* experiments. J-PS, CL, and TB designed the NMR and SPR experiments. EM, AP, PS, CL, TB, and AR wrote the manuscript. All the authors reviewed and approved the manuscript.

## Conflict of Interest

The authors declare that the research was conducted in the absence of any commercial or financial relationships that could be construed as a potential conflict of interest.

## Footnotes

1 http://www.nmr-bio.com/

## Supplementary Material

FIGURE S1 SPR of LptC–LptA_*m*_ interaction and its disruption by thanatin. **(A)** Determination of LptC–LptA_*m*_ dissociation constant. Left panel: Sensorgrams of LptC injected at different concentrations over immobilized LptA_*m*_. Right panel: Steady-state analysis of LptC–LptA_*m*_ interaction. **(B)** Raw Sensorgrams of the data presented in Figure 5B.
