# Supplementary figures and images for: Thanatin Impairs Lipopolysaccharide Transport Complex Assembly by Targeting LptC–LptA Interaction and Decreasing LptA Stability
Source: Front Microbiol. 2020 May 13;11:909. doi: 10.3389/fmicb.2020.00909 (PMC7237710; doi:10.3389/fmicb.2020.00909)

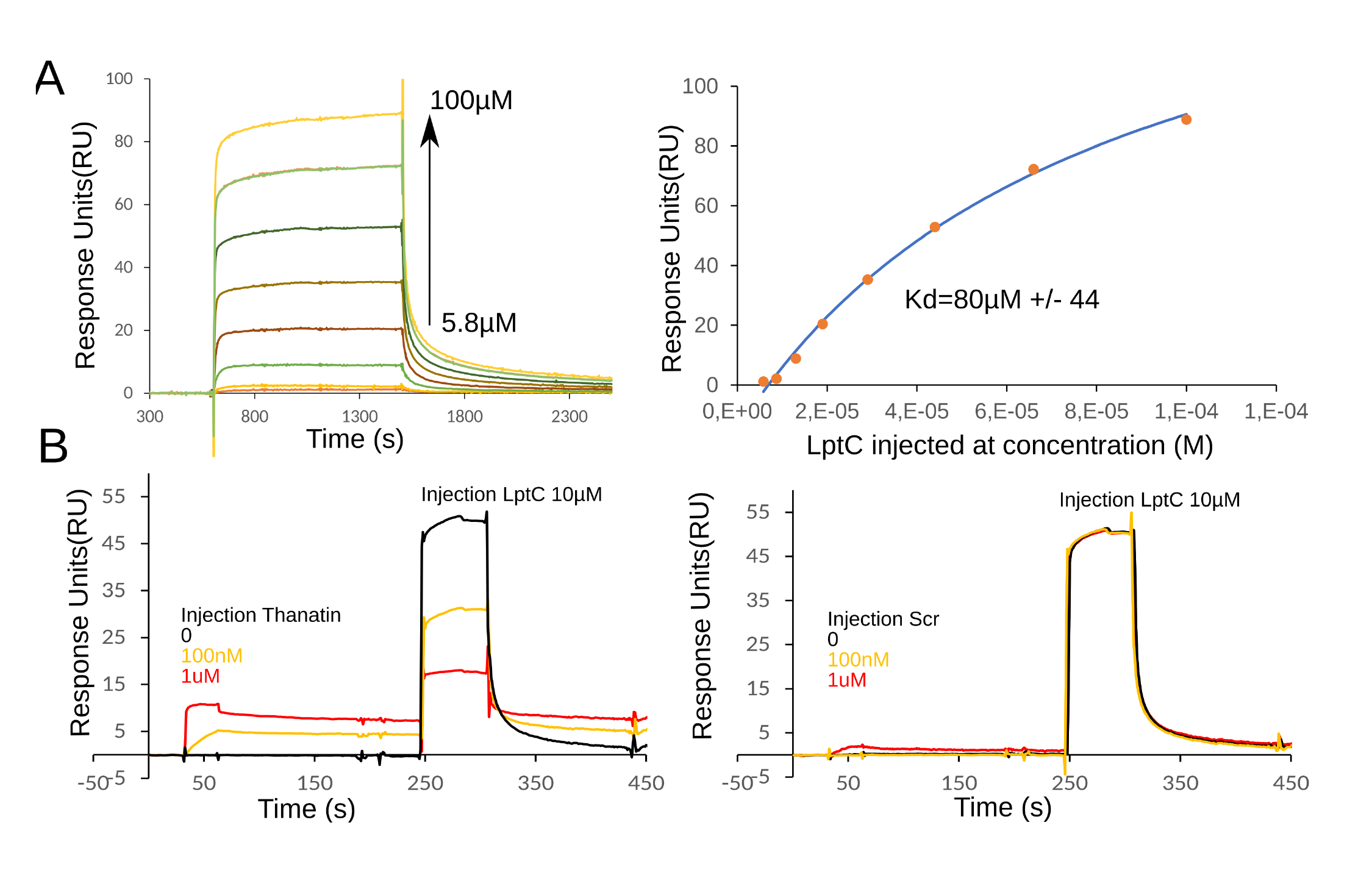

Supplement: FIGURE S1 — SPR of LptC–LptAm interaction and its disruption by thanatin. (A) Determination of LptC–LptAm dissociation constant. Left panel: Sensorgrams of LptC injected at different concentrations over immobilized LptAm. Right panel: Steady-state analysis of LptC–LptAm interaction. (B) Raw Sensorgrams of the data presented in Figure 5B. [file Image_1.TIF]
